# Indications for stenting of coarctation of the aorta in children under 3 months of age

**DOI:** 10.1007/s12471-020-01371-8

**Published:** 2020-02-13

**Authors:** T. Krasemann, I. van Beynum, M. Dalinghaus, W. van Leuwen, A. Bogers, P. van de Woestijne

**Affiliations:** 1 Department of Paediatrics, Division of Paediatric , Cardiology, Sophia Kinderziekenhuis, Erasmus MC Rotterdam, Rotterdam, The Netherlands; 2 Department of Cardiothoracic Surgery, Erasmus MC Rotterdam, Rotterdam, The Netherlands

**Keywords:** Aorta, Coarctation, Infant, Stenting

## Abstract

**Introduction:**

Coarctation of the aorta in children under 3 months of age is usually treated surgically. However, there are clinical scenarios in which stenting of native or recurrent coarctation may become necessary in this age group.

**Case reports:**

Four cases illustrate possible indications: left ventricular dysfunction increasing the operative risk, thrombus formation after coarctation surgery, patient size (i.e. in premature babies), and retrograde arch obstruction after hybrid palliation of hypoplastic left heart syndrome. In all babies, coarctation stenting was carried out successfully without complications.

**Conclusion:**

Coarctation stenting can be carried out safely in small children. Usually, the stent has to be removed or redilated later. Results are encouraging.

**Electronic supplementary material:**

The online version of this article (10.1007/s12471-020-01371-8) contains supplementary material, which is available to authorized users.

## What’s new?

- Stenting of (re-)coarctations can be carried out with good results even in very small children.
- The stents will have to be removed or redilated later.
- Angiography to delineate the anatomy can be carried out from the femoral sheath with good image quality.

## Introduction

In babies under 3 months of age, coarctation of the aorta is usually treated surgically in the first instance. However, there are scenarios in which alternative treatment is indicated either due to unfavourable results after initial surgery, or due to patient characteristics.

Stenting of coarctation of the aorta (CoA) is well established in adults and children weighing more than 25 kg, but even in smaller children this technique is gaining acceptance [1, 2]. In smaller children aged 3 months or more, ballooning is a treatment option for native and recurrent CoA [3], but there are recent reports of even smaller patients undergoing stent therapy as either a bridge to operation or as primary treatment [4–9]. Stenting of coarctations in small children has been carried out for several reasons in a limited number of patients [4–8, 10].

In patients undergoing a hybrid procedure for hypoplastic left heart syndrome or borderline left ventricles, stenting of the descending arch may become necessary to facilitate adequate reverse flow into the aortic arch, should the ductal stent cover the distal aortic arch [9].

We describe four cases of CoA stenting carried out in children under 3 months of age, in each one for a different reason. We think that these cases illustrate typical indications, which are: (1) severely impaired left ventricular function, where surgery as primary treatment carries a high risk; (2) postoperative thrombus formation within the operated area of the aorta; (3) prematurity in babies in whom surgery is postponed until a higher weight is reached; and finally (4) potential obstruction of the connection of the descending aortic arch to the ductal arch in patients after hybrid therapy for hypoplastic left heart syndrome and variants.

## Case reports

### Case 1

**Fig. 1:**
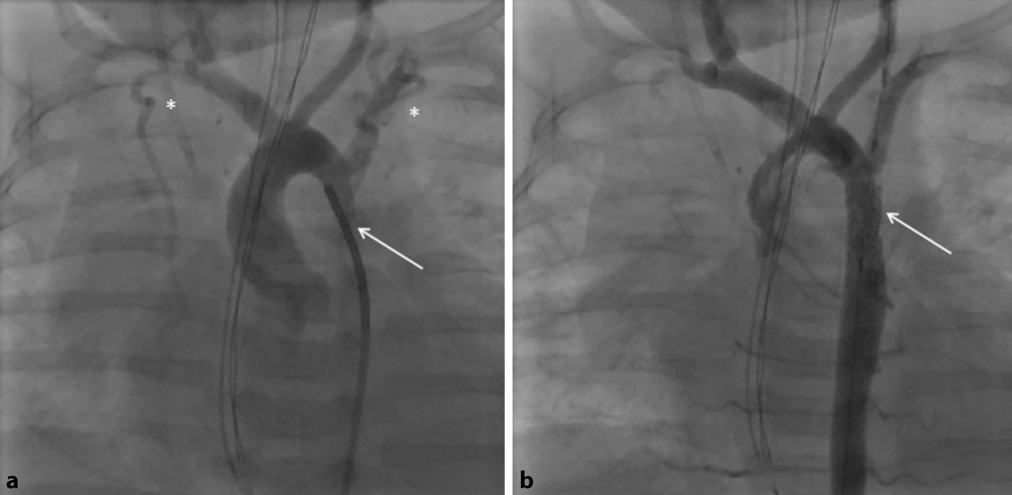
**a**, **b** Baby with severe re-coarctation 6 weeks after coarctectomy. **a** Injection into the aortic arch. The re-coarctation is completely obstructed by the 4F catheter (*arrow*). Collateral arteries are marked with *asterisks*. **b** With a Cook Formula stent in situ the lumen is unobstructed and continuity restored (*arrow*). Collateral flow is hardly visible anymore

A female patient, in whom CoA resection with aortic arch plasty with a homograft patch had been performed on day 10 of life, was admitted aged 2.5 months (5.3 kg) with severe (pinpoint) re-coarctation (Fig. 1a) and extremely poor function of the dilated left ventricle (fractional shortening 4%) on echocardiography (Electronic Supplementary Material, Movie 1). Dobutamine and adrenaline were started. Surgery was deemed high risk. The baby was brought immediately to the catheter laboratory. Ballooning of the re-coarctation had no effect. Via a 6F sheath, angiography was performed without an additional catheter, confirming severe re-coarctation. This was passed with a 0.014″ Ironman wire (Abbott, St Paul, MN, USA), and a short Cook Formula stent (6 × 12 mm) (Cook Medical, Bloomington, IN, USA) was implanted (Fig. 1b), abandoning the invasively measured blood pressure gradient of 80 mm Hg completely. Inotropic support could be weaned within 1 day. Within 2 days ventricular function recovered to a fractional shortening of 24% (Electronic Supplementary Material, Movie 2). No clinical problems occurred during follow-up, especially no access-related problems. Redilation is planned dependent on blood pressure gradients, but has not been necessary over 15 months following the procedure.

### Case 2

**Fig. 2:**
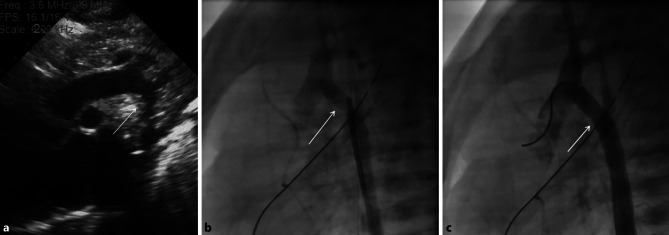
**a** Echocardiographic image of the aortic arch. Note the thrombus presenting as an irregular shape at the inner curvature, which is echodense. **b** Hand injection into the transverse arch. *Arrow* Less contrast in the distal arch representing thrombus (also confirmed on echocardiography). **c** Stent in the transverse arch, no residual thrombus visible

A female patient (3.3 kg) underwent coarctectomy with end-to-end anastomosis on day 14 of life. Postoperatively, thrombus formation occurred on the anastomosis site, which appeared on echocardiography as an echodense area of the inner curvature of the arch with an irregular surface (Fig. 2a). Redo surgery was performed, but was again followed by thrombus formation. Through a 4F sheath in the femoral artery, angiography was performed (Fig. 2b), confirming the thrombus in the transverse arch. A Resolute Onyx stent 4.5 × 8 mm (Medtronic, Fridley, MN, USA) was placed between the left carotid artery and the left subclavian artery, compressing the residual thrombus into the vessel wall (Fig. 2b). The child received low-molecular-weight heparin subcutaneously, until 2 months later the stent was removed surgically and an aortic arch plasty was carried out with a homograft patch. We did not observe access-related problems. Unfortunately, the child developed re-coarctation distal to the left subclavian artery 5 months later, which was treated with balloon dilation. Further follow-up was uneventful.

On careful review of all the data, the only variance from standard operative treatment was the use of a different aortic clamp than is usually used during surgery. However, it is not clear if this affected thrombogenicity inside the vessel, as no dissection flap was found during redo surgery or on angiography. Further laboratory testing did not reveal any thrombopathy, and the further course was uneventful in this respect.

### Case 3

**Fig. 3:**
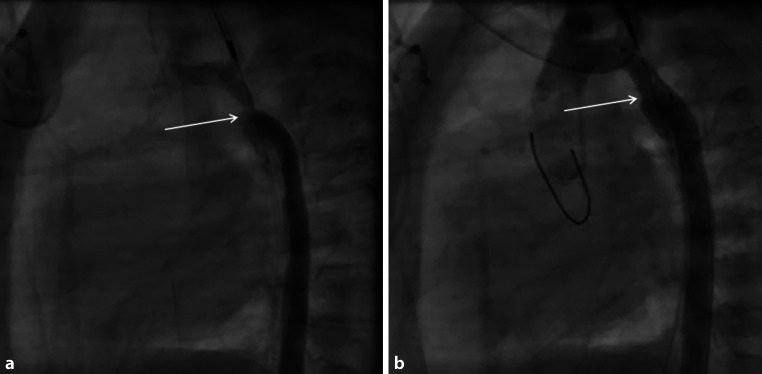
**a**, **b** Premature baby, native coarctation and hypoplasia of the descending arch (*arrow*). **a** Hand injection through the femoral sheath delineating the anatomy. **b** Onyx stent in situ. Note the expanded diameter of the isthmus with the stent (*arrow*)

A premature male of 33 weeks’ gestation, a second twin (Apgar scores 2/8/9, birthweight 1.715 kg), had coarctation with a concomitant perimembranous ventricular septal defect. Prostaglandin E infusion was initiated for 6 weeks to achieve further growth. An intervention was planned to facilitate surgery at a later stage. This was carried out at a corrected gestational age of 38 weeks (weight 2.2 kg). Via a 4F sheath, angiography was carried out, delineating the anatomy (Fig. 3a). An Onyx stent 4 × 8 mm (Medtronic) was implanted without a long sheath (Fig. 3b), abandoning the gradient of 50 mm Hg completely. Three months later the stent was removed and an extended end-to-end anastomosis was performed. The child is doing well 14 months later, with no residual or recurrent CoA and no access-related problems.

### Case 4

**Fig. 4:**
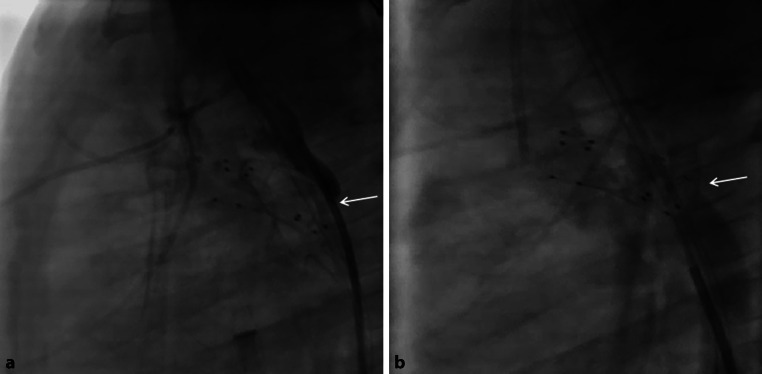
**a**, **b** Baby with borderline left ventricle, status after hybrid palliation. Forward flow through the aorta. **a** Catheter placed through the struts of the Sinusflex-DS stent, hand injection. The 4F catheter completely obstructs the lumen (*arrow*). **b** Onyx stent placed through the struts of the Sinusflex-DS stent. Hand injection into the descending aorta. Note the washout in the area of the newly placed stent (*arrow*), representing forward flow from the aortic arch

A 10-week-old girl weighing 4 kg with a borderline left ventricle (not apex-forming after birth, aortic valve diameter 5 mm, Z‑score −2.8) had undergone a hybrid approach with bilateral pulmonary artery banding and placement of a self-expanding stent (Sinusflex-DS, Optimed, Ettlingen, Germany) in the arterial duct at the age of 5 days, and an additional Sinusflex-DS stent due to constriction of the distal ductal arch at the age of 5 weeks. There was antegrade flow over the aortic arch, but on echocardiography the distal aortic arch at the connection to the ductal arch seemed stenosed with increasing flow velocity on conventional Doppler ultrasonography. Angiography confirmed the stenosed distal aortic arch (Fig. 4a). Through the struts of the Sinusflex-DS stent, a 0.014″ Ironman guidewire (Abbott) was forwarded into the aortic arch via a 5F TorqVue sheath for stabilisation. An Onyx stent 5 × 9 mm (Medtronic) was then carefully placed and deployed (Fig. 4b). Angiographically, flow through the distal aortic arch improved. Four weeks later, the baby underwent biventricular repair consisting of surgical removal of all stents, aortic arch plasty with homograft, closure of atrial and ventricular septal defects, and reconstruction of the right ventricular outflow tract. Five months after this operation, the aortic arch was redilated with an 8‑mm balloon. No further interventions were performed during the following year.

## Discussion

Coarctation stenting can be carried out safely in small children to facilitate surgery at a later stage, or for acute postoperative problems. Of course, technical considerations include the choice of sheaths, catheters and stents. The smallest sheath possible should be chosen to avoid access problems, which have been described previously [3]. Through the sheath, angiography even with dilute contrast can visualise the whole aorta, avoiding manipulation with a catheter. In children with a ductal stent in situ after hybrid palliation for hypoplastic left heart syndrome or a borderline left ventricle, a catheter placed closer to the stenosed aortic segment may be beneficial to delineate the anatomy in detail, and to avoid interference of the ductal arch filling with contrast.

Coronary guidewires like the Ironman we used in our case series are typically atraumatic and allow guidance of the balloon-crimped stent through the coarctation, giving enough support to the balloon-stent ensemble.

Stents can be coronary stents or small redilatable ones which can be deployed through small sheaths. We used Onyx stents in three of our patients. These are drug-eluting stents, which are frequently used in adult coronary interventions. At the time of our interventions, non-drug-eluting stents were not stocked (and hence not available). An ideal stent in this age group would be a bioabsorbable one which keeps its mechanical properties long enough to maintain vessel patency until, after absorption, no further scaffold is necessary. Unfortunately, no such stent exists yet. In our three patients treated with coronary stents, these were removed completely during further surgery. The Cook Formula stent can be redilated repeatedly, as necessary, according to patient size [10]. Redilation is not possible in self-expanding stents, which therefore are not recommended in this clinical setting. Procedural details of all procedures are summarised in Table 1.

**Table 1:** Demographic data and interventional information

Patient	Age (weeks)	Cardiac diagnosis	Extracardiac diagnosis	Weight at intervention (kg)	Sheath size (max, F)	Stent used	Fluoroscopy time (min)	Radiation dose (dose area product) (µGy m^2^)
1	10	CoA	n/a	5.3	6	Cook Formula 6 × 12 mm	6.9	109.8
2	2	CoA	n/a	3.3	4	Resolute Onyx 4.5 × 8 mm	4.0	32.6
3	5	CoA	Prematurity 33/40 weeks	2.2	4	Resolute Onyx 4 × 8 mm	3.0	11.1
4	10	CoA, borderline LV, s/p hybrid palliation	n/a	4.0	5	Resolute Onyx 5 × 9 mm	9.3	94.3

*CoA* coarctation of the aorta, *LV* left ventricle, *s/p* status post, *Hybrid palliation* bilateral pulmonary artery banding and ductal stent

Indications for stent placement in a coarctation in this age group vary, but this is mainly carried out to stabilise patients. Of course, one has to take into account that further interventions or operations are unavoidable, because of patient growth.

Our first case demonstrates how left ventricular function can be impaired by a severe (re‑)coarctation, and how this improved once the stent was placed. Depending on the duration of the impairment, recovery may take some time before the stent can be surgically removed [11]. In a previously described case, multi-organ failure recovered completely after coarctation stenting in a newborn, which could be operated successfully 4 weeks later [6]. We used a short stent, because during a coarctectomy typically a short aortic segment has to be removed anyway, and we did not want to extend the operation, i.e. by patch augmentation of the aorta.

Thrombus formation at the operation site of a coarctation is rare, and can be problematic. Compressing the thrombus into the vessel wall can be achieved, but the stent needs to be long enough to cover the whole thrombus. In our case, a stent of 8 mm length was sufficient. Anticoagulation may be necessary to avoid recurrent thrombus formation at the stent site. We opted for subcutaneous heparin, as oral anticoagulation in this age group can be challenging. Our knowledge to date indicates that this is the first patient in the literature to successfully undergo treatment for an acute thrombus after coarctation surgery with a stent. Comparable techniques have been used to treat thrombotic pulmonary artery occlusion, but in chronic settings [12].

Patient size may also be an indication; in these cases stent placement facilitates growth of the patient until a coarctectomy can be carried out safely. This has been done in children with a weight as low as 680 g [5]. Again, a short stent is preferable to make the operation as simple as possible; during coarctectomy the stented segment can simply be excised and end-to-end anastomosis performed. Operations can be delayed up to 2.5 years, and weight gain can be normalised until then [5].

In children with hypoplastic left heart syndrome who have undergone a hybrid procedure either as a bridge to a Norwood procedure or as the first step of staged palliation, ‘reverse’ coarctation with obstruction of the descending arch can cause problems both for the upper half of the body and coronary perfusion, in the case of retrograde perfusion [9]. In these cases, stenting can be performed through the side struts of a previously placed self-expandable Sinusflex-DS stent. Ballooning alone will not be sufficient, as the struts have a high tendency to recoil [13]. In our case, there was forward flow through the aortic arch, but the technical considerations are the same.

## Conclusion

Stenting of native or recurrent coarctations can be carried out even in young children under 3 months of age for various reasons and with good results. Attention needs to be paid to sheath size and the stent material used. If an operation is planned in due course, stents which are not redilatable can be used. Angiography can be carried out from the femoral sheath with good image quality.

## Caption Electronic Supplementary Material

**Movie 1**: Case 1. Long axis view of left ventricular function pre-intervention

**Movie 2**: Case 1. Long axis view of left ventricular function 3 days post-intervention
